# The influence of corporate social responsibility on safety behavior: The importance of psychological safety and the authentic leadership

**DOI:** 10.3389/fpubh.2022.1090404

**Published:** 2022-11-30

**Authors:** Byung-Jik Kim, Min-Jik Kim, Julak Lee

**Affiliations:** ^1^College of Business, University of Ulsan, Ulsan, South Korea; ^2^Department of Psychology, Yonsei University, Seoul, South Korea; ^3^School of Industrial Management, Korea University of Technology and Education, Cheonan, South Chungcheong, South Korea; ^4^Department of Industrial Security, Chung-Ang University, Seoul, South Korea

**Keywords:** corporate social responsibility, safety behavior, psychological safety, authentic leadership, moderated mediation model

## Abstract

Considering the importance of safety behavior, the current study investigates the relationship between CSR and safety behavior. To be specific, we delve into the underlying mechanism and its contingent factor of the association. This paper proposes that CSR promotes employee safety behavior through the mediation of psychological safety. In addition, authentic leadership may function as a positive moderator that amplifies the positive effect of CSR on psychological safety. Utilizing 3-wave time-lagged survey data from 213 South Korean workers, the current study empirically tests the hypotheses by establishing a moderated mediation model by utilizing structural equation modeling. The results demonstrate that CSR enhances employees' safety behavior by increasing their psychological safety and that authentic leadership positively moderates the relationship between CSR and psychological safety. This research's findings have meaningful theoretical and practical implications.

## Introduction

In the past few decades, corporate social responsibility (CSR) has emerged as a critical issue for scholars and practitioners. Although the concept has been described in different ways, the majority of researchers have agreed that CSR means various activities and policies that promote economic, social, and environmental benefits *via* satisfying stakeholder requirements (e.g., employees, customers, local communities, governments, and the environment) (1–5). Several works have reported that CSR is an effective strategy to enhance an organization's competitive advantage (3, 6–8) by enhancing its reputation or prestige (9), consumer evaluations of the firm and its products (10), its attractiveness to investors (11), and its financial performance (12, 13).

Specifically, regarding employees' responses toward CSR, extant studies have demonstrated that CSR improves employees' perceptions, attitudes, and behaviors, including organizational commitment and identification, work engagement, job satisfaction, creativity, innovative behavior, and helping behavior (6, 14–20).

As previously mentioned, although various CSR scholars have examined CSR's influence on organizational outcomes, research gaps still exist that must be complemented. First, extant works on CSR have underexplored CSR's influence on employee safety behavior (6, 15). According to Burke et al. (21), employee safety behavior is defined as “actions or behaviors that individuals exhibit in almost all jobs to promote the health and safety of workers, clients, the public, and the environment” (p. 432). Employee safety behavior has been recognized as a critical predictor of accidents and injuries at work (22). Scholars have especially emphasized its importance after the COVID-19 pandemic because it functions as a crucial antecedent of both customer and organizational safety during major health crises (23). Considering the significance of safety behavior in an organization, we need to investigate the influence of CSR on safety behavior.

Second, pertinent to the first research gap, there has been little research on the intermediating processes (i.e., mediators) and the contingent factors (i.e., moderators) of the link between CSR and safety behavior (6, 8, 15). Given that discovering the intermediating mechanisms and their contingent factors can help to systematically understand this relationship (6, 15), it is meaningful to investigate the mediators and moderators of the relationship between CSR and safety behavior.

Third, existing studies on CSR have paid relatively less attention to the important role of leadership, especially the leader's “authenticity” (6, 15, 24). A leader possesses a significant authority to make several critical decisions such as assigning tasks, evaluating performance, and establishing implicit norms in an organization, eventually critically affecting employees' perceptions, attitudes, and behaviors (25–27). In addition, from the point view of a follower, his or her leader is a symbolic figure who represents the organization itself, meaning that the employee is likely to perceive the leaders' purposes or behaviors as those of the organization (28, 29). Therefore, the employees are likely to consider the degree of their leaders' authenticity as their organizations' one. Based on the argument, we suggest that a leader's authenticity may function as a critical moderator in the process of firms' CSR activities, which is why it is important to examine how a leader's authentic behaviors affect and interact with CSR (24).

To fill the research gaps mentioned above, we examine the underlying mechanism and its contextual factors in the relationship between CSR and safety behavior. Although few studies have investigated CSR's influence on safety behavior, based on the social exchange theory, we can infer that CSR promotes employee safety behavior. According to social exchange theory, when one party supplies another party with support and benefits, the beneficiary will likely feel a sense of obligation to repay (30, 31). Employees are both direct and indirect beneficiaries of the organization's CSR activities because they are some of the most important stakeholders (6, 8, 15). Thus, they are likely to feel a sense of duty toward their organization, eventually repaying it through positive behaviors such as safety behavior (6, 32).

***Hypothesis 1:*** CSR may increase employee safety behavior.

Specifically, the current study suggests that employees' psychological safety mediates the association between CSR and their safety behavior. Moreover, authentic leadership functions as a positive moderating factor that amplifies CSR's positive influence on psychological safety.

First, the current paper proposes that CSR enhances employee psychological safety. Psychological safety can be defined as an employee's perception that he or she is safe to take risks or confront tough issues in the organization (33, 34). According to the basic concept of CSR (4, 5), CSR activities contain a variety of charitable acts, investments, and services for internal stakeholders (i.e., employees) as well as external stakeholders (e.g., local communities, the natural environment, and customers) (35). More specifically, CSR for employees includes various training, education, and safety programs for enhancing employees' capabilities, well-being, and safety (35). Through these practices, an employee will likely feel that he or she is supported and treated as a precious member of the organization. These positive experiences directly make the employee feel safe in the organization (26, 34, 36, 37).

In addition, CSR for local community members (e.g., government organizations, nongovernmental organizations, and the socially disadvantaged), the environment, and customers may indirectly influence employees' perceptions and attitudes toward their organization. When a firm proactively conducts its social responsibility for the various external stakeholders, the employees are likely to perceive that the organization is ethical and trustworthy. Then, this morally based trust toward the organization may diminish employees' anxiety about uncomfortable issues pertinent to them, encouraging them to feel less vulnerable (26, 34, 37, 38). Based on the above arguments, this research proposes that CSR activities boost employee psychological safety.

***Hypothesis 2:*** CSR may increase employee psychological safety.

Next, this research suggests that an employee's psychological safety enhances his or her safety behavior. To the best of our knowledge, there have been few studies that investigate the influence of psychological safety on safety behavior (34, 37, 39). However, based on the social exchange theory (30, 31), we expect that psychological safety promotes employee safety behavior (23, 34, 37). According to the social exchange perspective, an individual or a group tends to maintain balance in relationships, which is called “the rule of reciprocity” (31, 32). Thus, when an individual or a group is given precious things by someone or some group, the beneficiary is likely to feel a sense of obligation to repay it similarly (30, 32).

For instance, from an employee's point of view, experiencing psychological safety at work is likely to be perceived as a psychological reward (30, 34, 37). One of the primary reasons that the employee works in the organization may be for financial reward. However, he or she receives the reward in the form of a wage based on an official contract. In this situation, the employee is likely to perceive that the positive experiences gained *via* psychological safety are an additional reward beyond the contract. Then, the employee may feel a sense of duty to repay the additional reward from the organization. It is reasonable for the employee to repay by demonstrating positive attitudes or behaviors toward the organization, such as safety behavior (23, 32, 34). By increasing safety behaviors which correspond with achieving the organization's goals and success as well as diminishing unsafe behaviors that are incongruent with its direction, the employee may feel a sense of balance in the relationship with the organization (23, 32).

***Hypothesis 3:*** An employee's psychological safety may increase his or her safety behavior.

Then, to integrate the relationships among the research variables as described above (i.e., CSR, psychological safety, and safety behavior), the current paper suggests that employee psychological safety mediates the relationship between CSR and safety behavior. This mediation structure is supported by a context-attitude-behavior framework (38, 40). According to this perspective, an organization possesses several environmental and contextual factors such as systems, practices, rules, and climates, which substantially build members' attitudes and behaviors. CSR is a critical context that influences employees' attitudes, such as psychological safety, eventually building their behaviors, such as safety behaviors. Thus, we suggest that CSR affects employee safety behavior *via* the mediation of psychological safety.

***Hypothesis 4:*** An employee's psychological safety mediates the relationship between CSR and safety behavior.

Moreover, and more critically, we propose that authentic leadership is an important moderator which amplifies CSR's positive impact on psychological safety. As described above, our argument that CSR enhances employee psychological safety is reasonable. However, the link between CSR and psychological safety may not always be valid or applied equally to all situations, organizations, or individual employees, because there may be contextual or contingent variables (e.g., personality, values, motivational characteristics, leadership style, organizational climates, rules, and systems) that moderate the link in a real organization (34, 37).

Among several potential moderating variables, we focus on the role of the “authenticity' of a leader, which is reflected by the degree of authentic leadership from the perspective of followers. Authentic leadership has been defined as a leader's transparent and balanced decision-making patterns based on his or her internalized moral standards and self-awareness. In other words, authentic leadership indicates the degree of “authenticity' of the leader behaviors (41–43). Considering that not only employees are likely to perceive the organization as a “human-like” entity by providing it with humanlike characteristics, including various purposes and intentions (44), the employees may think that the leader symbolizes the organization itself. As a result, they are likely to consider the leaders' intentions or behaviors as those of the organization. Thus, they are likely to regard their leader's authenticity as a criterion to judge whether the activities and systems of the organization are authentic and genuine based on its unique values and philosophy (28, 29). In other words, from the perspective of followers, the degree of authentic leadership significantly represents the level of their organization's authenticity in implementing a variety of activities, practices, policies, and systems. For example, when a leader cannot show sufficient authentic leadership, employees are likely to believe that other hidden intentions or purposes may exist behind the moral acts.

Given that the authenticity of CSR functions as a critical criterion in realizing the positive impacts of the moral acts (45, 46), this doubt about the authenticity of CSR will decrease the positive influence of CSR on psychological safety (28). On the contrary, when authentic leadership is high, employees are likely to consider that the organization makes various decisions and strategies relying on its own value systems and philosophy. Then, the employees feel that the organization's CSR is trustworthy and authentic (28). Therefore, this paper suggests that authentic leadership may positively moderate the link between CSR and psychological safety.

***Hypothesis 5:*** Authentic leadership may function as a positive moderator which amplifies the enhancing effect of CSR on psychological safety.

**Figure 1:**
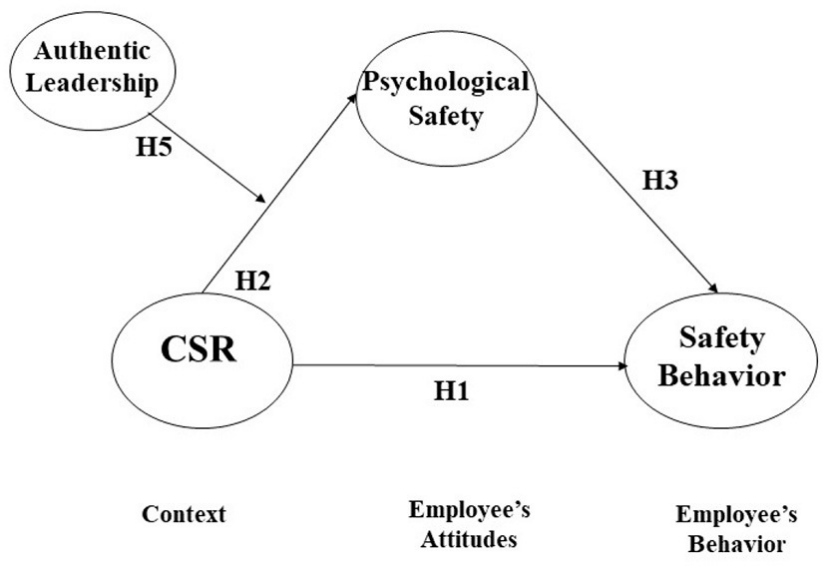
Theoretical model.

Taken together, this paper examines CSR's impact on safety behavior *via* the mediating effect of psychological safety. In addition, the current study proposes that authentic leadership may function as a positive moderator which amplifies the positive effect of CSR on psychological safety (see Figure 1). To test our hypotheses, this research established a moderated mediation model with structural equation modeling (SEM) based on 3-wave time-lagged data from 213 Korean employees. The current paper positively contributes to both the CSR and safety behavior literature as follows. First, the current paper aims to reveal CSR's influence on employee safety behavior, considering that the link between CSR and safety behavior has been underexplored despite its importance to an organization.

Second, this research investigates the intermediating mechanism (i.e., a mediator) and its contextual factor (i.e., a moderator) of the relationship between CSR and safety behavior. Third, we emphasize the critical role of leadership by demonstrating that a leader's authenticity functions as a positive moderator which amplifies the enhancing effect of CSR on psychological safety. Lastly, from a methodological perspective, we aim to complement the limitations of cross-sectional research design by applying a longitudinal approach (i.e., a 3-wave time-lagged design).

***Hypothesis 1:*** CSR may increase employee safety behavior.
***Hypothesis 2:*** CSR may increase employee psychological safety.
***Hypothesis 3:*** An employee's psychological safety may increase his or her safety behavior.
***Hypothesis 4:*** An employee's psychological safety mediates the relationship between CSR and safety behavior.
***Hypothesis 5:*** Authentic leadership may function as a positive moderator which amplifies the enhancing effect of CSR on psychological safety.

## Methods

### Participants and procedure

This study's participants consisted of employees over 20 years old who currently work in various organizations in South Korea. We gathered the data across three different time points. We recruited the participants through an online survey company which has an online survey system with the largest population of panelists, totaling about 3,450,000. The participants reported their occupation status when they registered for the system *via* a user authentication function (e.g., a cell phone number or email address). Online survey systems have been recognized as a trustworthy method for accessing various samples (47).

The current study collected data from employees in South Korean firms from three different time points. By collecting data through three different time periods, this study attempts to complement the fundamental issues embedded in cross-sectional research design. The online system's operating functions enabled us to track who responded to our survey, verifying that the participants through the three time points were identical. The time interval between first survey and second one was either 4 or 5 weeks, and the interval between second one and last one was 12 or 13 weeks. The reason why the time intervals in between T1–T2, and T2–T3 are different is that the influence of CSR on an employee's “behaviors” needs more time to be unveiled in compared to the impact of CSR on his or her “attitudes' (6, 15, 20). Our survey system was available for 2 or 3 days at each time point to provide enough time for participants to respond. When the system was available, participants could approach it whenever they wanted. The company monitored the integrity of the data using traps for geo-IP violators and timestamps to flag efficient responding that restricted participants from logging into the survey site and completing the surveys multiple times.

The research company's experts contacted the participants to obtain their permission to participate in the survey, assuring not only that their participation would be voluntary but also that their responses would be confidential and used only for research purposes. Further, the company reported and obtained both the informed consents and compliance with ethical requirements from those who agreed with the participation and reporting. The research firm granted the respondents a financial reward (US $8). This research was approved by the IRB (Institutional Review Board) of one of the representative universities in South Korea.

The research company randomly chose the participants in a stratified way to diminish the possibility of sampling bias. In stratified sampling, a random sample is drawn from each of the strata. Through the stratified random sampling method, the biases from several employee characteristics that were likely to affect the results (e.g., gender, age, position, education, and industry type) were diminished.

During Time Point 1, 407 employees responded to our survey; at Time Point 2, 299 workers responded to the second survey; and at Time Point 3, 217 employees responded to the third survey. After collecting the responses, we eliminated the missing data. Finally, we utilized data from 213 participants who submitted complete responses to all three-wave surveys (response rate: 52.33%). To determine the sample size, we utilized various suggestions from previous research. First, we confirmed whether our sample size was proper by calculating the minimum sample size with G^*^Power version 3.1.9.7. A power analysis with the program demonstrated that a sample size of 213 provided sufficient power (≥0.70) to detect a medium effect with an alpha level of *p* = 0.05 (48). In addition, Barclay et al. (49) proposed that one observable variable requires at least 10 cases (i.e., the rule of 10) in conducting a structural equation modeling analysis. Because the research model has 22 observable variables, the final 213 cases are proper. The participants' features are displayed in Table 1.

**Table 1:** Descriptive characteristics of the sample.

**Characteristic**	**Percent**
**Gender**	
Male	52.6%
Female	47.4%
**Age (years)**	
20–29	12.7%
30–39	33.8%
40–49	32.4%
50–59	21.1%
**Education**	
Below high school	9.9%
Community college	18.8%
Bachelor's degree	59.6%
Master's degree or higher	11.7%
**Occupation**	
Office worker	71.4%
Profession (practitioner)	7.5%
Production worker	5.2%
Public official	4.2%
Administrative positions	4.2%
Sales and Service	2.3%
Education	0.5%
Freelance	0.5%
Others	4.2%
**Position**	
Staff	23.0%
Assistant manager	18.3%
Manager or deputy general manager	33.8%
Department/general manager or director	24.9%
and above	
**Tenure (years)**	
Below 5	49.8%
5–10	22.5%
11–15	14.6%
16–20	6.5%
21–25	2.4%
Above 26	4.2%
**Industry type**	
Manufacturing	23.0%
Construction	13.2%
Wholesale/Retail business	11.7%
Health and welfare	10.8%
Information service and telecommunications	8.9%
Education	8.5%
Services	6.6%
Financial/insurance	2.8%
Consulting and advertising Others	0.9%
Others	9.4%

### Measures

Each time point's survey measured distinct variables of our research model. At Time Point 1, the respondents were asked about the level of CSR and authentic leadership. At Time Point 2, the participants' data were gathered to measure their perceived degree of psychological safety. At Time Point 3, we collected data about participants' safety behavior. These variables were assessed through multi-item scales on a five-point Likert scale (1 = strongly disagree, 5 = strongly agree). Moreover, through Cronbach alpha values, we computed the internal consistency of each variable.

### CSR (time point 1, collected from employees)

We measured the degree of CSR in each organization using 12 items of Turker's CSR scale. This measure was developed by applying a stakeholder perspective that concentrates on CSR acts for numerous stakeholders. In the current study, we choose four dimensions of CSR practices: environment, community, employee, and customer. The four dimensions include three items. In the case of the environment dimension, the sample item was “our company participates in activities which aim to protect and improve the quality of the natural environment.” Regarding the community dimension, the sample item was “our company contributes to campaigns and projects that promote the well-being of the society.” Regarding the employee dimension, the sample item was “The management of our company is primarily concerned with the employees' needs and wants.” Regarding the customer dimension, the sample item was “our company respects consumer rights beyond the legal requirements.” The Cronbach's alpha value is 0.90.

### Authentic leadership (time point 1, collected from employees)

To assess the degree of authentic leadership, we utilized twelve items (Cronbach alpha = 0.96) from the Authentic Leadership Inventory (ALI) developed and validated by Neider and Schriesheim (42). ALI has been known to possess a high content, convergent, and discriminant validity (42, 50). We asked respondents to measure the authenticity of their immediate leaders at Time Point 1. ALI consists of four dimensions: (1) self-awareness (e.g., “The leader is clearly aware of the impact he/she has on others”), (2) relational transparency (e.g., “The leader expresses his/her ideas and thoughts clearly to others”), (3) internalized moral perspective (e.g., “The leader is guided in his/her actions by internal moral standards”), and (4) balanced processing (e.g., “The leader carefully listens to alternative perspectives before reaching a conclusion”). ALI has been acknowledged as a valid scale, as verified by previous research (50). The Cronbach's alpha value was 0.91.

### Psychological safety (time point 2, collected from employees)

We evaluated the degree of employee psychological safety by utilizing four items from a psychological safety scale developed by Edmondson (33). This scale measures an employee's perception of psychological safety. Sample items were “it is safe to take a risk in this organization,” “I am able to bring up problems and tough issues in this organization,” “it is easy for me to ask other members of this organization for help,” and “no one in this organization would deliberately act in a way that undermines my efforts.” These items were used in previous studies with South Korean employees [e.g., 16]. The Cronbach's alpha value was 0.82.

### Safety behavior (time point 3, collected from employees)

To evaluate the degree of employee safety behavior, six items developed by Neal and Griffin (51) were utilized. This measure consists of two sub-dimensions: three items for safety compliance, and three items for safety participation. The sample item for safety compliance is “I use all the necessary safety equipment to do my job.” The sample item for safety participation is “I voluntarily carry out tasks or activities that help to improve workplace safety and so on.” The Cronbach's alpha value was 0.89.

### Control variables

Considering the suggestions of previous studies (22, 23), this research controlled for safety behavior by utilizing several control variables such as tenure, gender, position, and education. The variables were gathered at Time Point 2.

### Statistical analysis

First, we performed a frequency analysis to check the participants' demographic features. We conducted a Pearson correlation analysis with the SPSS 26 program to compute the relationships among our research variables. Then, according to the suggestion of Anderson and Gerbing (52), we took a two-step approach that consists of (1) the measurement and (2) the structural model. To test the validity of the measurement model, we performed a Confirmatory Factor Analysis (CFA). Next, based on SEM, a moderated mediation model analysis with the maximum likelihood (ML) estimator was performed with the AMOS 23 program to test the structural model.

To test whether the various model fit indices are acceptable, this study utilized a variety of goodness-of-fit indices including the comparative fit index (CFI), the Tucker–Lewis index (TLI), and the root mean square error of approximation (RMSEA). Extant research has reported that CFI and TLI values >0.90 as well as an RMSEA value <0.06 are proper (53). Next, a bootstrapping analysis was implemented to test whether the indirect effect was significant (54). Lastly, to check whether our mediation hypothesis is supported, we conducted a bootstrapping analysis with a 95% bias-corrected confidence interval (CI). This analysis tests the significance of the indirect mediating effect. If the CI does not include zero (0), this indicates that the indirect effect is statistically significant at a 0.05 level (54).

## Results

### Descriptive statistics

Our research variables, such as CSR, authentic leadership, psychological safety, and safety behavior, are significantly associated. The correlation analysis results are shown in Table 2.

**Table 2:** Correlation between research variables.

	**Mean**	**S.D**.	**1**	**2**	**3**	**4**	**5**	**6**	**7**
1. Gender_T2	1.47	0.50	–						
2. Education_T2	2.73	0.79	−0.18**	-					
3. Tenure_T2	7.91	7.57	−0.32**	−0.06	-				
4. Position_T2	3.04	1.62	−0.46**	0.24**	0.26**	-			
5. CSR_T1	3.20	0.62	−0.22**	0.08	0.18**	0.14*	-		
6. AL_T1	3.22	0.59	−0.10	0.01	0.03	0.14*	0.37**	-	
7. PS_T2	3.24	0.59	−0.25**	0.10	0.16*	0.28**	0.37**	0.31**	-
9. SB_T3	3.71	0.56	−0.20**	0.05	0.12	0.14*	0.30**	0.22**	0.31**

* *p* < 0.05.

** *p* < 0.01. S.D. means standard deviation, CSR means corporate social responsibility, AL means authentic leadership, PS means psychological safety, and SB indicates safety behavior. As for gender, males are coded as 1 and females as 2. As for position, general manager or higher are coded as 5, deputy general manager and department manager 4, assistant manager 3, clerk 2, and others below clerk as 1. As for education, “below high school diploma” level is coded as 1, “community college” level as 2, “bachelor's” level as 3, and “master's degree or more” level is coded as 5.

### Measurement model

To test the discriminant validity of the main research variables (CSR, authentic leadership, psychological safety, and safety behavior), we performed a CFA for all items by checking the measurement model's goodness of fit. Specifically, we compared our hypothesized model, a four-factor model (CSR, authentic leadership, psychological safety, and safety behavior), to other alternative models such as three-, two-, and one-factor models by conducting a series of chi-square difference tests.

First, the hypothesized that the four-factor model has a good and acceptable fit [χ^2^ (d*f* = 109) = 172.988; CFI = 0.963; TLI = 0.954; RMSEA = 0.053]. Then, we conducted a series of chi-square difference tests by comparing the four-factor model to a three-factor model [χ^2^ (df = 112) = 378.522; CFI = 0.848; TLI = 0.815; RMSEA = 0.106], a two-factor model [χ^2^ (df = 114) = 802.601; CFI = 0.606; TLI = 0.530; RMSEA = 0.169], and a one-factor model [χ^2^ (df = 115) = 876.954; CFI = 0.564; TLI = 0.485; RMSEA = 0.177]. The results of the chi-square difference tests indicated that the four-factor model was better than the others. Thus, this result indicates that our four research variables have a proper degree of discriminant validity.

### Structural model

We constructed a moderated mediation model that includes both mediation and moderation structures in the link between CSR and safety behavior. In the mediation structure, the link between CSR and safety behavior is mediated by the degree of employee psychological safety. In the moderation structure, authentic leadership functions as a positive moderator which amplifies the positive impact of CSR on psychological safety.

Next, in the moderation structure, we multiplied the two variables (i.e., CSR, authentic leadership) to create an interaction term between the variables. Before multiplication, the two variables were centered on their means to decrease the harmful impact of multi-collinearity. Such a centering method increases the validity of the moderation analysis by not only diminishing the degree of multi-collinearity between the variables but also minimizing the loss of correlations (55).

To test the impact of the multi-collinearity bias, we measured the value of the variance inflation factors (VIF) and tolerance (55). The VIF values for CSR and authentic leadership were 1.157 and 1.157, respectively. Moreover, the values of tolerance were 0.864 and 0.864, respectively. These results with VIF values smaller than 10 and tolerance values above 0.2 indicate that CSR and authentic leadership are relatively free from the multi-collinearity issue.

### Results of mediation analysis

To find the best mediation model, we compared a full mediation model to a partial mediation model by performing a chi-square difference test. The full mediation model is identical to the partial mediation model except for the direct path from CSR to safety behavior. The fit indices of both the full mediation model [χ^2^ = 215.459 (df = 131), CFI = 0.941, TLI = 0.923, and RMSEA = 0.055] and the partial mediation model [χ^2^ = 209.148 (df = 130), CFI = 0.945, TLI = 0.928, and RMSEA = 0.054] were acceptable. However, the chi-square difference test between the models (Δχ^2^ (1) = 6.311, *p* < 0.05) demonstrated that the partial mediation model was superior, indicating that CSR is likely to directly and indirectly influence (e.g., *via* the mediating effect of psychological safety) safety behavior rather than directly impact it.

The control variables, such as tenure, gender, education, and position, were included in the research model to control for the dependent variable, safety behavior. The results show that all the control variables were not statistically significant. Including the control variables, our research model demonstrates that CSR is significantly associated with employee safety behavior (β = 0.22, *p* < 0.01), supporting Hypothesis 1. For Hypothesis 1, the coefficient value of the path from CSR to safety behavior was in the “partial” mediation model (which was superior to the full mediation model) that was finally accepted. This result is consistent with the fact that the model fit indices of partial mediation are better than those of full mediation. Based on the results of the chi-square difference test between the full mediation model and partial mediation model as well as the significant value of the path coefficient, we conclude that Hypothesis 1 is supported. In other words, CSR is likely to influence safety behavior in a both direct and indirect way through the mediating effects of various mediators (e.g., psychological safety).

**Figure 2:**
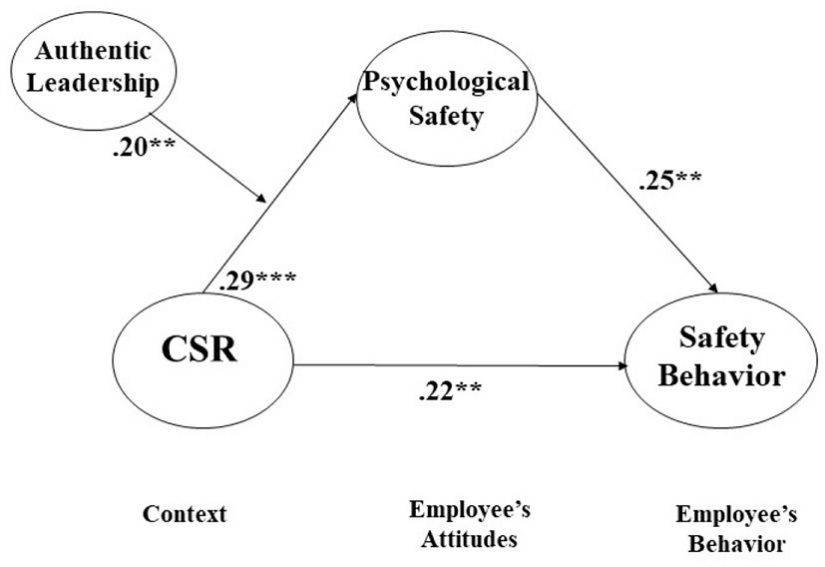
Coefficient values of our research model (** *p* < *0.01*, *** *p* < *0.001*. All values are standardized).

Further, CSR is significantly and positively associated with the employees' psychological safety (β = 0.29, *p* < 0.001), supporting Hypothesis 2, and psychological safety is significantly and positively associated with their safety behavior (β = 0.25, *p* < 0.01), supporting Hypothesis 3 (see Table 3 and Figure 2).

**Table 3:** Results of structural model.

**Hypothesis**	**Path (relationship)**	**Estimate**	**S.E**.	**Standardized** **estimate**	**Supported**
1	CSR -> safety behavior	0.177	0.068	0.218**	Yes
2	CSR -> psychological safety	0.241	0.067	0.289***	Yes
3	Psychological safety -> safety behavior	0.240	0.081	0.246**	Yes
5	CSR × authentic leadership	0.303	0.111	0.196**	Yes

** *p* < 0.01,

*** *p* < 0.05. Estimate indicates standardized coefficients. S.E. means standard error.

### Bootstrapping

To test psychological safety's mediating effect on the link between CSR and safety behavior (Hypothesis 4), we conducted a bootstrapping analysis with a sample size of 10,000 (54). The indirect mediation effect is significant at the 5% level if the 95% bias-corrected confidence interval (CI) for the effect of mean indirect mediation excludes 0 (54). The results indicate that the bias-corrected CI for the mean indirect effect did not include 0 (95% CI = [0.015, 0.162]), meaning that psychological safety's indirect mediation effect was statistically significant, supporting Hypothesis 4. The direct, indirect, and total effects of the paths from CSR to safety behavior are shown in Table 4.

**Table 4:** Direct, indirect, and total effects of the final research model.

**Model (Hypothesis 5)**	**Direct effect**	**Indirect effect**	**Total effect**
CSR -> Psychological Safety -> Safety Behavior	0.218	0.071	0.289

All values are standardized.

### Result of moderation analysis

**Figure 3:**
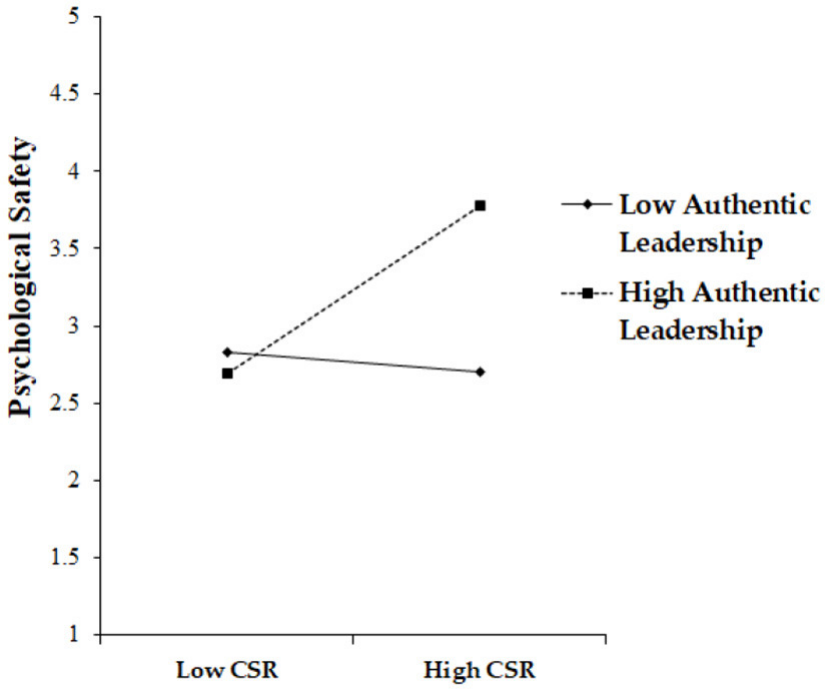
Moderating effect of authentic leadership in the CSR–psychological safety link.

We tested the moderating effect of authentic leadership on the relationship between CSR and psychological safety, which included conducting a mean-centering process by creating an interaction term. The coefficient value of the interaction term (β = 0.20, *p* < 0.01) was statistically significant. This result means that authentic leadership positively moderates the relationship between CSR and psychological safety by playing an amplifying role, indicating that when authentic leadership is high, CSR's enhancing impact on psychological safety can increase, supporting Hypothesis 5 (see Figure 3).

## Discussion

Utilizing 3-wave time-lagged data, this paper unveils that an employee's psychological safety is an underlying mechanism (i.e., mediator) in the relationship between CSR and safety behavior. The current paper also finds that authentic leadership positively moderates the relationship between CSR and psychological safety. The results are consistent with the previous literature on CSR, psychological safety, safety behavior, and authentic leadership. To be specific, CSR increased employee psychological safety (26, 34), and psychological safety promotes employee safety behavior (23, 26, 34). And this mediation structure is supported by a context-attitude-behavior framework (38, 40). Moreover, authentic leadership may function as a positive moderator which amplifies the enhancing effect of CSR on psychological safety (28, 29). Based on the results, we can conclude that our arguments can be supported from the theoretical and empirical perspectives.

The current paper can contribute to expanding the CSR and safety behavior literature by identifying a mediator and moderator that explain why and when CSR increases employee safety behavior. In the following part, we discuss the theoretical and practical implications and limitations and suggestions for future works.

### Theoretical implications

The current research positively contributes to the CSR literature from a theoretical perspective. First, by theoretically and empirically validating the underlying mechanism and its contextual factors in the link between CSR and safety behavior, we demonstrate why and when CSR significantly influences employee safety behavior. The importance of safety behavior has been increasingly emphasized after the COVID-19 pandemic because it is a critical antecedent of both customer and organizational safety during major health crises (23). However, there have been few studies on safety behavior despite its significance. Thus, this paper is helpful for scholars and practitioners to thoroughly understand CSR's influence on safety behavior (6, 15).

Second, we demonstrate that leadership is a crucial contextual variable that positively moderates the relationship between CSR and psychological safety. A leader tends to have substantial power to make several crucial decisions including assigning tasks, evaluating performance, and establishing implicit and explicit norms in an organization. Thus, the leader can significantly affect employees' perceptions, attitudes, and behaviors (25–27). Further, from an employee perspective, the leader is a symbolic actor of the organization itself, meaning that the employee may consider the intentions or behaviors of the leader as those of the organization (28, 29). Thus, our attempt to reveal the importance of leadership in the context of CSR is theoretically meaningful.

Third, this paper demonstrated the significance of authenticity, which is unveiled by a leader's behaviors, based on the empirical result that a leader's authentic behaviors positively moderate the link between CSR and psychological safety. No matter how proactively an organization conducts CSR activities, the positive influence of CSR on employee psychological safety is likely to diminish when there is not sufficient authenticity in the organization's moral acts (28, 45, 46). From the perspective of the employees, the organization's real intention in implementing the moral acts is critical to evaluate whether the moral behaviors of the organization are authentic. Considering the crucial role of a leader in evaluating the authenticity of CSR activities, the degree of authentic leadership can be the criterion to evaluate the degree of authenticity of CSR (28, 46).

### Practical implications

Our research provides practically meaningful implications to top management teams. First, this paper suggests that top management teams should consider CSR activities as an effective investment instead of merely a cost. Our results demonstrate that CSR activities increase employee safety behavior by enhancing their sense of psychological safety. Considering that both employees' sense of psychological safety and their safety behavior positively contribute to improving organizational outcomes, sincere and authentic attempts to implement CSR practices can be helpful to achieve the organization's goals and success.

Second, our results propose that top management teams should understand the essential role of leaders in conducting CSR activities. From the point of view of employees, the leader is a symbolic actor of the organization itself as well as a powerful authority to make several critical decisions within an organization. Thus, they are likely to consider the intentions or behaviors of the leader as those of the organization (28, 29). Top management teams should monitor and manage whether leaders properly affect in implementing CSR activities.

Lastly, top management teams need to understand the significance of authenticity that is reflected through authentic leadership. The positive influence of CSR activities on employees' psychological safety is amplified when they feel that the organization's CSR is authentic. However, on the contrary, when the degree of authenticity that is reflected in the level of authentic leadership is low, employees are less likely to feel psychological safety, indicating that authentic leadership substantially affects the positive influence of CSR activities on employees' attitudes (28, 45, 46). From the perspective of employees, the degree of authentic leadership is an important criterion to evaluate whether the organization's moral activities are genuine. Thus, because of the significance of authentic leadership, top management teams should cultivate authentic leadership in an organization (28).

### Study limitations and directions for future research

This paper has limitations that should be complemented. First, this paper could not adequately accommodate the cultural differences between Eastern and Western societies pertinent to how members perceive CSR in an organization. According to previous studies, Western cultures tend to emphasize the significance of socially imposed duties including CSR activities; therefore, the members are likely to be more sensitive to social obligations (56, 57). Because this paper gathered data only from employees in South Korea, we should cautiously interpret the results when applying them to other cultural contexts (56, 58). Although the spirit of CSR has been found to be universal (56), South Korean workers may respond to the call for CSR differently compared to Western workers. Therefore, further studies should properly consider this issue.

Second, this paper could not utilize an objective measure in evaluating the degree of CSR activities, as it only used subjective measures from employees. Although extant works on CSR have reported that subjective measures, including an employee's perception of CSR, can properly evaluate the real phenomena of CSR practices [e.g., (59)], future studies are required to utilize both types of measures and compare the different effects of each measure.

Third, this study could not sufficiently accommodate the discriminatory influence of the several sub-factors of CSR activities. As described above, the targets of CSR activities vary widely, including CSR for employees, consumers, local communities, and the environment. However, the current paper measured only four sub-constructs of CSR practices (i.e., CSR for employees, customers, society, and the environment). Thus, respondents may respond differently to the different targets (28, 60). For instance, Farooq et al. (35) demonstrated the differential influences of CSR on employees' perceptions by differentiating the CSR practices into internal CSR and external CSR. This issue must be adequately complemented in future research.

Fourth, the current paper could not adequately consider that utilizing control variables such age and gender as categorical variables, may have very little likely that it gets significant level. Thus, to complement the limitation, future studies should conduct other thorough analysis techniques such as multi-group analysis or Mann–Whitney U test that might offer different perspectives.

Fifth, although we utilized 3-wave time-lagged data, this research cannot be free from the issue of common method bias (CMB). To decrease this concern, we additionally conducted the Harman's single-factor test, that is the most widely utilized technique to evaluate CMB (61). The result demonstrated that merely 26.79% of covariance is explained by a single factor, meaning that the CMB issue was not serious. Nevertheless, future studies should validate the findings by utilizing multiple data sources.

## Conclusion

The current paper delves into CSR's impact on employee safety behavior. According to the results, CSR promotes employee safety behavior *via* the mediating role of psychological safety. Furthermore, authentic leadership functions as a positive moderator in the link between CSR and psychological safety. The results indicate that employee psychological safety is an underlying mechanism in translating CSR into safety behavior. Moreover, authentic leadership functions as an amplifying factor that enhances CSR's positive influence. Although this research has some limitations, we anticipate that these findings positively contribute to expanding the CSR literature.

## Data availability statement

The raw data supporting the conclusions of this article will be made available by the authors, without undue reservation.

## Ethics statement

The studies involving human/animal participants were reviewed and approved by Macromill Embrain Group of Ethics Committee. Macromill Embrain Group is the company providing market research service and their approval is sufficient according to the local requirements. The patients/participants provided their written informed consent to participate in this study.

## Author contributions

B-JK contributed by writing the original draft of the manuscript and in the conceptualization, data collection, formal analysis, and methodology. JL and M-JK contributed in the conceptualization, analysis, revision, and in editing the manuscript. All authors have read and agreed to the published version of the manuscript.

## Funding

This study was supported by the BK21 FOUR Program (Education and Research Center for Securing Cyber-Physical Space) through the National Research Foundation (NRF) funded by the Ministry of Education of Korea (5199990314137).

## Conflict of interest

The authors declare that the research was conducted in the absence of any commercial or financial relationships that could be construed as a potential conflict of interest.

## Publisher's note

All claims expressed in this article are solely those of the authors and do not necessarily represent those of their affiliated organizations, or those of the publisher, the editors and the reviewers. Any product that may be evaluated in this article, or claim that may be made by its manufacturer, is not guaranteed or endorsed by the publisher.
